# Effectiveness and Durability of a Quaternary Ammonium Compounds-Based Surface Coating to Reduce Surface Contamination

**DOI:** 10.3390/biology12050669

**Published:** 2023-04-28

**Authors:** Teresa Bento de Carvalho, Joana Bastos Barbosa, Paula Teixeira

**Affiliations:** Universidade Católica Portuguesa, Laboratório Associado, CBQF—Centro de Biotecnologia e Química Fina, Escola Superior de Biotecnologia, Rua Diego Botelho 1327, 4169-005 Porto, Portugal; s-mtmcarvalho@ucp.pt (T.B.d.C.); pcteixeira@ucp.pt (P.T.)

**Keywords:** antimicrobial activity, foodborne pathogens, quaternary ammonium compounds, contact surfaces

## Abstract

**Simple Summary:**

The occurrence of cross-contamination of food in household kitchens could lead to severe foodborne diseases, so safe food practices are imperative. The study of the effectiveness and durability of a commercial quaternary ammonium compound-based surface coating regarding its antimicrobial efficacy, killing contact time, durability, and cytotoxicity was the objective of this study. Despite not showing cytotoxicity (if trace amounts of ≤0.2 mg/kg migrate into food when in contact with the surface) and being effective against three important pathogens in three artificially contaminated surfaces, its durability was less than one week on all surfaces cleaned in the usual manner. The regular use of quaternary ammonium compound-based surface coatings may significantly reduce surface contamination and increased food safety in domestic kitchens.

**Abstract:**

Foodborne diseases are of major concern as they have a significant impact on public health, both socially and economically. The occurrence of cross-contamination of food in household kitchens is a serious threat and the adoption of safe food practices is of paramount importance. This work aimed to study the effectiveness and durability of a commercial quaternary ammonium compound-based surface coating which, according to the manufacturer, retains its antimicrobial activity for 30 days, and is suitable for all types of hard surfaces for the prevention and/or control of cross-contamination. For that, its antimicrobial efficacy, killing contact time and durability on three different surfaces—polyvinyl chloride, glass, and stainless-steel—against three pathogens—*Escherichia coli* ATCC 25922, *Acinetobacter baumannii* ESB260 and *Listeria monocytogenes* Scott A—were tested according to the current antimicrobial treated surfaces efficacy test (ISO22196:2011). The results showed that the antimicrobial coating was effective against all pathogens with a reduction of >5.0 log CFU/cm^2^ in less than one minute for the three surfaces, but its durability was less than one week on all surfaces cleaned in the usual manner. Additionally, trace amounts (≤0.2 mg/kg) of the antimicrobial coating, which may migrate into food when contacting the surface, did not show cytotoxicity to human colorectal adenocarcinoma cells. The suggested antimicrobial coating has the potential to significantly reduce surface contamination, ensure surface disinfection and reduce the likelihood of cross-contamination in domestic kitchens, although it is less durable than suggested. The use of this technology in household settings is an attractive complement to the existing cleaning protocols and solutions that are already in place.

## 1. Introduction

Foodborne diseases have a significant social and economic impact on public health and place a heavy burden on healthcare systems.

From production to consumption, food undergoes several processes with control points needed to ensure food safety. If any of these required control points fail, foodborne illnesses are a likely outcome. Numbers from the most recent European Union One Health zoonosis report, released by the European Food Safety Authority (EFSA) and the European Centre for Disease Prevention and Control (ECDC), showed that in 2021, 34.1% of the total strong-evidence outbreaks occurred in households. Since not all household outbreaks are reported, this number is probably underestimated. *Campylobacter* and *Salmonella* caused most cases, while *Listeria monocytogenes* was linked to most hospitalisations and deaths [1,2].

Improper food handling in household kitchens represents a significant risk of transmission of foodborne pathogens due to cross-contamination from the contact of raw foods with hands, surfaces, cutting boards, utensils, and even ready-to-eat foods that may occur during food preparation [3,4]. However, this can be avoided with the use of safe food handling practices, personal hygiene, and proper cleaning of all surfaces and kitchen utensils that may come into contact with food [5].

The prevention of cross-contamination episodes that may lead to microbial foodborne diseases could be effectively carried out by using antimicrobial surface coatings that do not allow microorganisms to adhere to the surface and/or inhibit their growth [6]. The technology behind antimicrobial surfaces has been developed over the last few years, being introduced mainly in healthcare settings to complement pre-existing hygiene protocols to prevent infections and thus diminish the use of antibiotics to help fight ever-increasing antimicrobial resistance [7]. In the context of the COVID-19 pandemic which has been a daily burden since first emerging in early 2020, antimicrobial coatings could potentially lower the risk of transmission within healthcare and household settings and possibly work as a precautionary public health measure in regards to the prevention of future outbreaks [8,9].

There are two main mechanisms when it comes to how effective antimicrobial surface coatings work. Its function may rely on anti-adhesive proprieties that do not allow bacterial adhesion to surfaces, or on bactericidal proprieties, that kill the bacteria before or after contact with the surface. A combination of both mechanisms is also widely used to achieve optimal results [10]. The preeminent types of antimicrobial surface coating are antimicrobial peptides, antibacterial enzymes, nanoparticle-containing coatings such as gold or silver, quaternary ammonium compounds (QACs), anti-adhesive polymers via super hydrophobic coatings, and chitosan-based coatings [10]. Chitosan-based coatings already possess antibacterial properties, but additional antimicrobial compounds, such as QACs, are usually added to increase the biocidal effect. Their biocompatibility and ease of modification make them attractive components in the development of antimicrobial coatings [10]. Quaternary ammonium compounds are widely used in disinfectants as their primary mechanism of action is bacterial cell lysis. Disinfectants based on QACs are considered relatively safe and not harmful to humans if concentrations are kept within the values established by the Environmental Protection Agency (EPA).

The main objective of this work was to investigate the ability of a commercial QACs-based surface coating to inhibit important pathogens. To this end, the inhibition of *Escherichia coli* ATCC 25922, *Listeria monocytogenes* Scott A, and *Acinetobacter baumannii* ESB260 was evaluated when in contact with a commercial QAC-based coating on commonly used surfaces of polyvinyl chloride, glass, and stainless-steel. In addition, the durability and microbial inactivation kinetics of the coating on each surface were also tested, as well as its cytotoxic activity in vestigial concentrations on relevant target cells.

## 2. Materials and Methods

### 2.1. Coating Compound and Surfaces Used in This Study

The antimicrobial coating (solution) (biocidal active substance: <0.5% *w*/*w* Dimethyloctadecyl ammonium chloride; <0.3% *w*/*w* Didecyldimethylammonium chloride; <0.2% *w*/*w* Quaternary ammonium compounds, benzyl-C12-16-alkyldimethyl, chlorides; excipients and solvents 100%. Other: Disinfectants) was purchased from a local supermarket on the basis that it was compatible with all surfaces, that it would retain antimicrobial activity for 30 days, and that it could be used for an array of settings from the household to hospital and public transports, to deter and control cross-contamination of hard surfaces.

Slides of glass, polyvinyl chloride (PVC), and stainless steel measuring approximately 25 mm × 75 mm were used. Prior to antimicrobial activity assays, each surface was sterilised using 70% (*v*/*v*) bleach, washed three times with deionised sterile water to remove residual bleach and impurities, and dried at 60 °C overnight in sterile Petri dishes to ensure an aseptic environment. Following the manufacturer’s recommendations, the antimicrobial coating was then applied to the surfaces by aerosolisation, ensuring an even distribution of the product throughout the surface, and allowed to dry. 

For each surface, testing was performed on nine specimens (surfaces), three treated and six untreated.

### 2.2. Preparation of Test Inoculum

*Escherichia coli* ATCC 25922, *L. monocytogenes* Scott A and *A. baumannii* ESB260 (all available in the culture collection of Escola Superior de Biotecnologia, Porto, Portugal), stored at −80 °C in Trypto-Casein Soy Broth with Yeast Extract (0.6% *w*/*v*) (TSBYE; Biokar Diagnostics, Beauvais, France) containing 30% (*v*/*v*) of glycerol (Sigma, Steinheim, Germany) were sub-cultured twice before use in assays. Each isolate was cultured in Trypto-Casein Soy Agar with Yeast Extract (0.6% *w*/*v*) (TSAYE; Biokar diagnostics, Beauvais, France) at 37 °C for 18 h. Then, one single colony of each isolate was transferred to TSBYE (Biokar), incubated at 37 °C for 18 h, and this culture was subsequently diluted at 1:100 in TSBYE and incubated in the same conditions to achieve optimal cell density (confirmed by plate enumeration). After incubation, cells were centrifuged at 8131 rcf for 5 min (Eppendorf Mini spin, Hamburg, Germany), washed, and resuspended at the initial volume in diluted nutrient broth (NB, Biokar Diagnostics). Serial dilutions were performed to achieve the required bacterial level (ca. 10^5^ CFU/mL). 

### 2.3. ISO22196:2011 Measurement of Antibacterial Activity on Plastics and Other Non-Porous Surfaces

Antimicrobial activity was measured in accordance with the International Organization for Standardization (ISO) protocol 22196 [11] standard (International Standard Organization, 2011) but with modifications. Sterile surfaces were placed in Petri dishes and inoculated with 200 μL of the test inoculum. Sterile plastic pieces measuring 15 mm × 65 mm were used to cover the test inoculum and gently pressed down to the edges of the film, without any leaking to the edge of the surface. Immediately after inoculation, the recovery of bacteria from three untreated specimens was determined by washing the inoculated surface with 10 mL of Dey-Engley (D/E) neutraliser (BD Difco, NJ, USA), which was used as the initial suspension ten-fold diluted. This suspension was serially diluted in 1:500 NB, and 100 μL of each dilution was spread on Plate Count Agar (PCA, Biokar Diagnostics) and incubated for 48 h at 37 °C. 

Half of the inoculated untreated specimens and the inoculated treated specimens were incubated at 22 °C, for 24 h, and Petri dishes were covered with damp sterile gauze to achieve a relative humidity of no less than 90% to prevent drying of the inoculum. Recovery of the viable bacteria was performed as mentioned above.

After incubation, plates containing 30 to 300 colonies were counted, and the number of viable bacteria in CFU/cm^2^ was determined according to the standard parameters for test validation. Three independent replicates were performed.

#### 2.3.1. Antimicrobial Coating Kinetics

The inactivation kinetics of the antimicrobial coating at 22 °C were assessed according to ISO22196 [11], as previously described in 2.3, but at different time points (1, 10 and 20 min). Each bacterial inoculum was exposed to the antimicrobial coating at exposure times of 1, 10 and 20 min, at 22 °C (room temperature). Three independent replicates were performed. 

#### 2.3.2. Antimicrobial Coating Durability

The durability of the antimicrobial activity of the coating on the different surfaces was measured according to ISO22196 [11]. Surfaces were treated with the antimicrobial coating as previously described. Antimicrobial activity measurement and surface cleaning were carried out on the day of application. The surfaces treated with the antimicrobial coating were lightly cleaned using four different methods, taking into account real-life scenarios of surface use and cleaning of high-touch surfaces in household kitchens: (i) wiped with bleach (concentration of 100% (*v*/*v*), (ii) wiped with a wet gauze cloth, (iii) wiped with a commercial surface disinfectant (Benzalkonium chloride 2% w/w, Isopropyl alcohol 8% *w*/*w*, excipients and water), and (iv) wiped with a commercial degreaser (contains among other ingredients: Anionic and non-ionic surfactants: 5% to 15%, phosphates: less than 5%. Contains potassium hydroxide) commonly used to clean surfaces. These cleaning processes were applied only once. The durability of the antimicrobial activity of the coating was assessed by contaminating the surface with the inoculum and performing the standard ISO protocol after the coating application and after the cleaning process. Three independent replicates were performed.

### 2.4. Cytotoxicity–MTT Assay

Human Colorectal Adenocarcinoma Cells Exposure to Vestigial Concentrations of Antimicrobial Test Coating

Cultured immortalised human colorectal adenocarcinoma (Caco-2) cells (ATCC ECACC 86010202, Manassas, VA, USA), at late post-confluence, were seeded in a 96-well microplate with a cell concentration of 9.5 × 10^4^ cells/mL in Dulbecco’s Modified Eagle’s Medium (DMEM, Lonza, Verviers, Belgium) with a glucose content of 4.5 g/L, supplemented with 10% (*v*/*v*) fetal bovine sera (FBS), 1% (*v*/*v*) Penicillin-Streptomycin-Fungizone solution (A/A), 1% (*v*/*v*) non-essential amino-acids (Gibco, Grand Island, NE, USA) and 1% (*v*/*v*) pyruvate (Gibco), and incubated for 24 h at 37 °C with an atmosphere containing 5% CO_2_. Cells were exposed to vestigial concentrations of the antimicrobial coating, 0.2 to 0.006 mg/kg, based on the EU Standing Committee on The Food Chain and Animal Health (Residues) statutory maximum residue level (MRLs) of 0.1 mg/kg for QACs, and incubated at the same conditions. A positive control (cells in the growth medium), negative control (cells with 30% (*v*/*v*) DMSO), and background control (medium only) were included. After incubation, the medium was discarded, and the MTT solution, composed of DMEM and MTT, was added to each well, and the plate was incubated for 2 h in the dark. The MTT solution was discarded, DMSO was added, and the plate was shaken for 15 min in the dark and at room temperature. Two independent replicas were carried out.

The absorbance of all the wells was measured at 570 nm using a microplate spectrophotometer (BioTek Synergy H1 Multimode Reader, Santa Clara, CA, USA), and the results were analysed using the following equation:

$$\%\;\mathrm{Metabolic}\;\mathrm{inhibition}=\frac{Abspositivecontrol-Abssample}{Abspositivecontrol}$$

### 2.5. Statistical Analysis

The average and standard deviation data were calculated using Microsoft Office TM 365 Excel 2016 (Microsoft, Redmond, WA, USA). Any statistically significant differences between means were assessed by an ANOVA test followed by a post-hoc test (Tukey HSD) calculated in IBM^®^ SPSSTM–IBM Statistic Analytics 28.0 (IBM, Chicago, IL, USA). The mean difference was considered significant at the 0.05 level.

## 3. Results

### 3.1. Measurement of Antibacterial Activity and Kinetics

**Figure 1:**
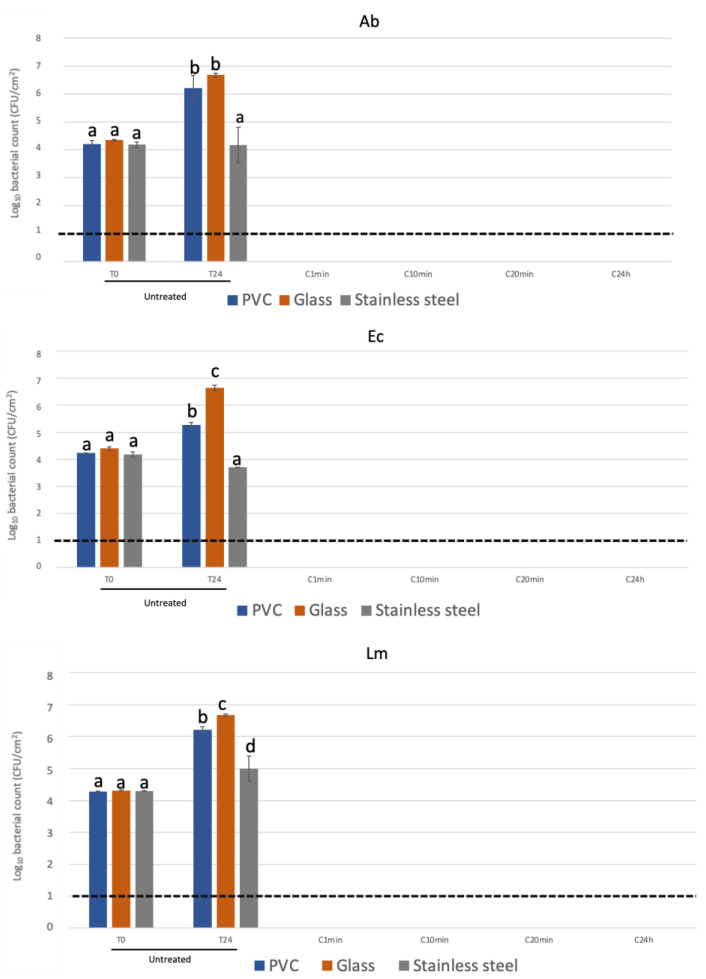
Contact killing time for *A. baumannii* ESB260 (Ab); *E. coli* ATCC 25922 (Ec) and *L. monocytogenes* Scott A (Lm). Recovery of bacteria immediately after inoculation (T0) and after 24 h incubation (T24) on untreated surface. Recovery of bacteria immediately after inoculation (C1 min), after 10 min (C10 min), 20 min (C20) and 24 h incubation (C24 h) on each treated surface. The results are means based on data from three replicates, and standard deviations are indicated by error bars. Equivalent lowercase letters mean no significant differences between each condition (*p* > 0.05). The dotted line indicates the detection limit of the enumeration technique.

The results obtained for the quaternary ammonium-based coating against each pathogen are shown in Figure 1.

Regarding the untreated surfaces, except for the stainless-steel surfaces for *A. baumanni* and *E. coli* where counts were equal to T0, bacterial growth was observed after 24 h (T24) for all surfaces and all pathogens. All pathogens were reduced to below the detection limit of the enumeration technique (1.0 log CFU/cm^2^) on all treated surfaces after 1 min of exposure.

### 3.2. Antimicrobial Activity Durability

**Figure 2:**
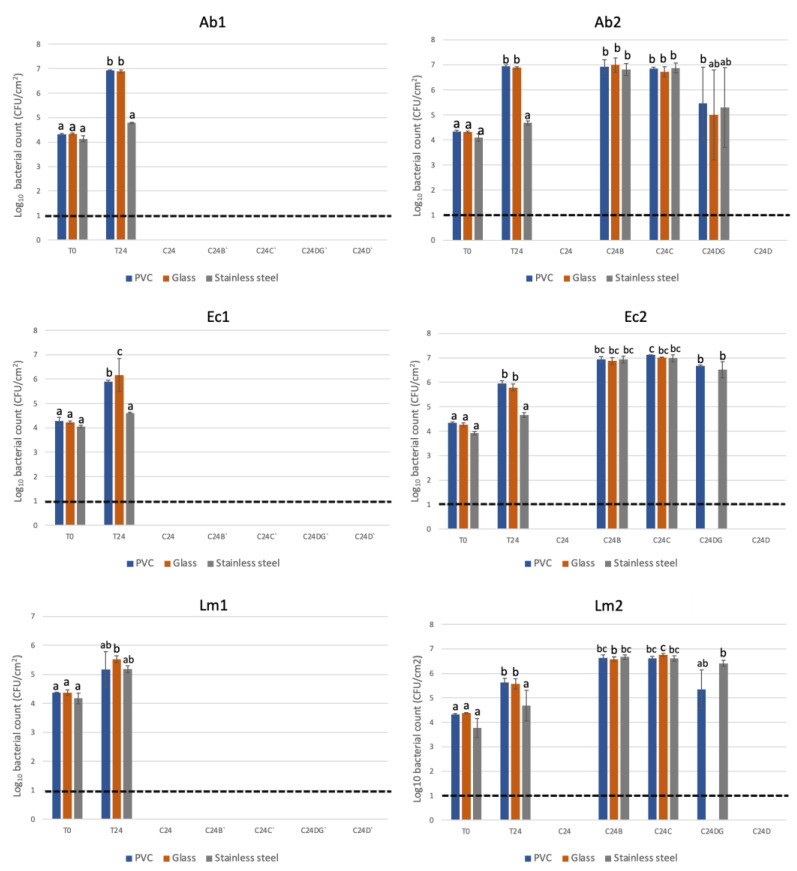
Recovery of bacteria immediately after inoculation (T0) and after 24 h incubation (T24) on untreated surfaces; and after 24 h incubation (C24 h; C24B’; C24C’; C24DG’ and C24D’) on each treated surface on day 1 for *A. baumannii* ESB260 (Ab1), *E. coli* ATCC 25922 (Ec1) and *L. monocytogenes* Scott A (Lm1). Antimicrobial activity of the coating on day 7 for *A. baumannii* ESB260 (Ab2), *E. coli* ATCC 25922 (Ec2) and *L. monocytogenes* Scott A (Lm2) with the recovery of bacteria immediately after inoculation (T0) and after 24 h incubation (T24) on untreated surface; and after 24 h incubation on treated surface cleaned with bleach (C24B); damp cloth (C24C); commercial degreaser (C24DG) and commercial disinfectant (C24D). The results are means based on data from three replicates, and standard deviations are indicated by error bars. Equivalent lowercase letters mean no significant differences between each condition (*p* > 0.05). The dotted line indicates the detection limit of the enumeration technique.

The antimicrobial activity of the coating against *A. baumannii* ESB260, *E. coli* ATCC 25922 and *L. monocytogenes* Scott A was evaluated on days 1 and 7 after surface cleaning with common household cleaning products (bleach, damp cloth, commercial degreaser and commercial disinfectant). The results are shown in Figure 2.

Despite the manufacturer’s claim of a 30-day antibacterial activity, only until day 7 was antimicrobial activity observed against all pathogens for all surfaces cleaned with the tested product (C24D). For *E. coli* and *L. monocytogenes*, no bacterial growth was also observed for glass surfaces cleaned with the commercial degreaser (C24DG).

### 3.3. Cytotoxicity of Caco-2 Cells Exposed to Vestigial Antimicrobial Coating Concentrations

**Figure 3:**
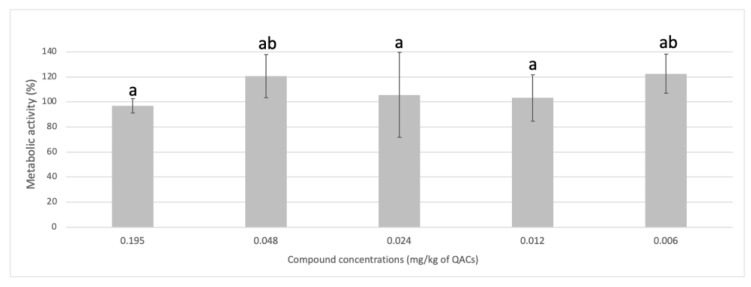
Metabolic activity of Caco-2 cells exposed to trace concentrations of the antimicrobial coating. The results are means based on data from two replicates, and standard deviations are indicated by error bars. Different letters indicate values significantly different (ANOVA test followed by Tukey HSD post-hoc, *p* < 0.05).

Figure 3 shows the results from the exposure of Caco-2 cells to trace concentrations of the antimicrobial test coating. Cells were able to proliferate when exposed to ≤0.2 mg/kg of the test compound.

## 4. Discussion

### 4.1. Measurement of Antibacterial Activity and Kinetics

The effectiveness of an antimicrobial coating was investigated in scenarios that mimicked real-life conditions. Surfaces commonly used in domestic kitchens, PVC, glass and stainless-steel, were used. The results from this study are in agreement with the previously published literature, being that the contact killing time was established at equal or less than 1 min after exposure to the treated surface, suggesting rapid and total growth inhibition in a short period of exposure [12,13]. Furthermore, contact-killing-based coatings have a clear advantage compared to other types of antimicrobial coatings, mainly due to their stability on the surface, high and broad-spectrum antimicrobial activity, the relative unlikeliness of antimicrobial resistance, and high durability on surfaces [14]. However, it is important to state that, regarding antimicrobial surfaces, the risk of spreading multi-drug resistance lies not only in developing resistance to a specific antimicrobial agent but also in the occurrence of two phenomena, cross-resistance, and co-resistance. Cross-resistance happens when a single molecular mechanism is capable of mediating resistance to more than one antimicrobial agent. Co-resistance can occur when mechanisms encoding resistance or reduced susceptibility are genetically linked; thus, the selective pressure conferred by the presence of one antimicrobial co-selects for the other and ensures the retention of all of these within the population [7,8].

The antibacterial activity of quaternary ammonium compounds (QACs) against *E. coli* and *L. monocytogenes* has been reported by several authors [15,16,17,18,19,20,21]. However, some authors have also reported that some QAC formulae were ineffective against *E. coli* [21,22]. Regarding *A. baumannii*, Ramzi et al. [21] and Reichel et al. [23] have reported that different formulations had an impact on the antibacterial activity of the QAC-based coatings [21,23]. QAC-based coatings are known to have good antibacterial activity towards Gram-positive bacteria and enveloped viruses [14,24]. From the view of disinfection, they are bactericidal for a wide range of pathogens such as *E. coli*, *Staphylococcus aureus*, *Streptococcus mutans*, *Bacillus subtilis*, *Pseudomonas aeruginosa* and the fungal pathogen *Candida albicans* [25,26,27,28]. QACs were initially used in common liquid disinfectant solutions, but they are currently studied for use in antimicrobial-coated surfaces [27,29]. This is due to the fact that these compounds have been proven to be very stable, mostly unaffected by pH, easily dissolved in water, do not evaporate, and solutions containing QACs, when dry, leave a solid residue that allows prolonged activity on contact surfaces for a long time [10,27,29]. Nevertheless, they can also be effective towards Gram-negative bacteria; *E. coli* ATCC 25922 was inhibited by the antimicrobial QAC-based coating tested (Figure 1). Multiple factors regarding biocide formulation have been shown to influence its antibacterial effect. The molecular weight of the QACs used is the most reported factor because of its direct impact on the efficacy of the compound and its cytotoxicity [30,31].

Several reports show the high antimicrobial activity of QACs, with their efficacy being proven for a myriad of applications, ranging from clinical settings to food-related facilities and household environments [17,32].

### 4.2. Antimicrobial Activity Durability

QAC-based surface coatings have been reported to be long-lasting disinfection alternatives [33]. Testing of the antimicrobial coating durability, meaning the durability of antimicrobial activity of the agent on relevant surfaces such as PVC, glass and stainless-steel, was assessed one time a week over 30 days due to the claim of a 30-day antimicrobial efficacy by the manufacturer. However, no antimicrobial activity was observed after 7 days. The fact that only *A. baumannii* was able to grow on the glass surface cleaned with the degreaser may be related to its adept ability to form biofilms, which may have played a role in the survival and bacterial growth on the surface [34]. Environmental aspects, such as pH, temperature, and the hydrophobicity or hydrophilicity of the surface, are also essential influencers for bacterial adhesion [35]. The low durability of the tested coating goes against several reports of the long-term antimicrobial durability of QAC-based coatings (Figure 2). However, this may be due to certain disinfectants disturbing the coating layer and inactivating it, or physical removal of the coating layer itself. In a study with viruses by Butot et al. [36], the authors showed that the antiviral activity of a QAC-based coating was removed after only one cleaning round. The authors proposed that the coating sprayed onto the surface was probably removed by cleaning during the cleaning process [36].

With the high efficacy of killing both Gram-positive and Gram-negative pathogens, for common day-to-day household use, treated surfaces should be solely cleaned with a dry cloth to avoid disrupting the coating layer and consequently impairing the antibacterial efficacy. To ensure disinfection and no pathogen survival, it is recommended that reapplication of the coating must be performed every week.

One of the main shortcomings of ISO22196 is that testing was carried out in non-real-life scenarios and under optimal artificial conditions. Thus, an overestimation of the efficacy of the antimicrobial agent may be caused by test parameters such as high incubation temperature, high relative humidity, bacterial concentration, and contact time that do not reflect what happens in clinical or food processing settings, making it difficult to extrapolate any results [37].

### 4.3. Cytotoxicity of Caco-2 Cells Exposed to Vestigial Antimicrobial Coating Concentrations

Even though a myriad of antimicrobial agents commonly used can migrate from antimicrobial-treated surfaces to food matrices, cytotoxicity assays are highly important to be carried out due to the possible unintentional ingestion [6].

The statutory maximum residue level (MRLs) of 0.1 mg/kg for two of the most used quaternary ammonium compounds, didecyldimethylammonium chloride (DDAC) and benzalkonium chloride (BAC), was voted through at the EU Standing Committee on The Food Chain and Animal Health (Residues) in 2014. Thus, the concentrations were chosen including this limit (0.195–0.006 mg/kg). Trace amounts of the test compound were shown to be non-cytotoxic toward Caco-2 cells, with cells showing proliferation when exposed to the concentrations tested (Figure 3). These results can be explained by the hormesis effect in which low doses of a particular compound have a beneficial effect. In this case, the stimulation effect of low concentrations of the antimicrobial coating induces cell proliferation, whereas higher concentrations induce a toxic effect on the cells. Hormesis is considered an adaptive process following homeostasis disruption caused by overcompensation for mild stress [38,39,40].

Antimicrobial compound migration to food products is considered contamination and a failure within the coating composition due to the premise that the active coating should remain fully attached to its substrate, i.e., the food contact surface treated with the antimicrobial agent. The probability of food contamination by antimicrobial migration and subsequently lowered durability on the surface are two reasons why antimicrobial coatings are still a rare find within commercial applications [6,41].

Trace amounts of cleaning products in food depend on the amount of product required to be used in surface disinfection, the effectiveness of removal methods after treatment, and the ratio between the surface area to volume of the food in contact with the surface. High ratios, such as porous and/or creviced and cracked surfaces, have the most potential to transfer trace amounts of disinfectants [42,43]. In addition, migration of the antimicrobial coating on the food contact surface to food products can occur if, after disinfection, a residue of the antimicrobial agent used is not adequately removed from the treated surface [44].

Toxicology reports for disinfectants used on food contact surfaces are required to assess better which antimicrobial agent may be used for differing situations. Monitoring, approval, and maximum residue levels may differ from country to country [6].

## 5. Conclusions

In conclusion, the commercial antimicrobial surface QAC-based coating might be a good solution to decrease cross-contamination events in household kitchens. The coating was proven to have antimicrobial activity, fully inhibiting bacterial recovery after a 1 min contact time on treated surfaces for three relevant pathogens, *A. baumannii* ESB260, *E. coli* ATCC 25922 and *L. monocytogenes* Scott A, on PVC, glass and stainless-steel surfaces. Regarding the durability of the product on the surfaces and maintenance of antimicrobial activity after cleaning, it was shown to be less than that reported by the manufacturer (<7 days). However, antimicrobial activity can be guaranteed by complementing the everyday cleaning with a commercial disinfectant or applying the coating more frequently than every 7 days. Furthermore, vestigial concentrations of antimicrobial coating (<0.2 mg/kg) were not cytotoxic to Caco-2 cells, which is indicative of its safety in case of ingestion of a food product onto which the coating has been transferred.

Although promising, with a significant reduction in surface contamination, the studied commercial antimicrobial coating should be further evaluated, extending the study to more pathogens and surfaces and even to surfaces contaminated with environmental contaminants and with other pathogens simultaneously. In addition, the efficacy of this antimicrobial coating when the pathogens are incorporated within a food matrix as well as with possible acquired antimicrobial resistance should be the focus of further studies.

## Data Availability

Not applicable.
